# Supplementary material for: Uptake of monoaromatic hydrocarbons during biodegradation by FadL channel-mediated lateral diffusion
Source: Nat Commun. 2020 Dec 10;11:6331. doi: 10.1038/s41467-020-20126-y (PMC7728783; doi:10.1038/s41467-020-20126-y)
Supplement: Supplementary file 1 — Supplementary Information [file 41467_2020_20126_MOESM1_ESM.pdf]

Supplementary Information

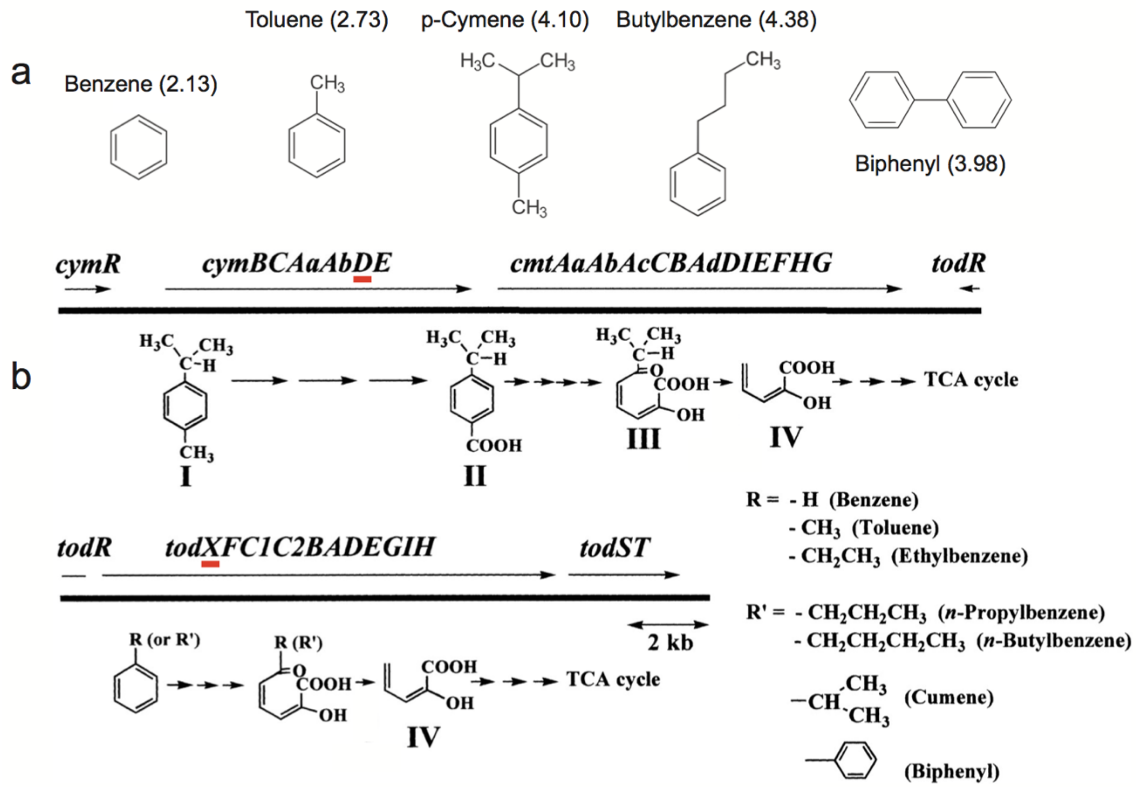

Supplementary Figure 1 Substrates and biodegradation operons of Pp F1. a, PpF1 AH substrates with membrane partition coefficients (log Kp values) listed in parentheses. Butylbenzene and biphenyl are degraded by mutant strains of PpF1 with an extended substrate range that have mutations in *cymR* as well as in *todS*. The result of the first mutation is constitutive expression of CmtE, which has an analogous function to TodF but with a broad substrate range. The mutation in TodS results in induction of the *tod* operon by the new substrates. Thus, both operons work together in the degradation of extended-range growth substrates such as biphenyl[1]. **b**, Organization of the *cym*/*cmt* (top) and *tod* operons (bottom) on the PpF1 genome, and illustration of various enzymatic reactions during MAH biodegradation. The locations of *cymD* and *todX* are highlighted in red. *todR* is a non-functional, LysR-type regulator[2].

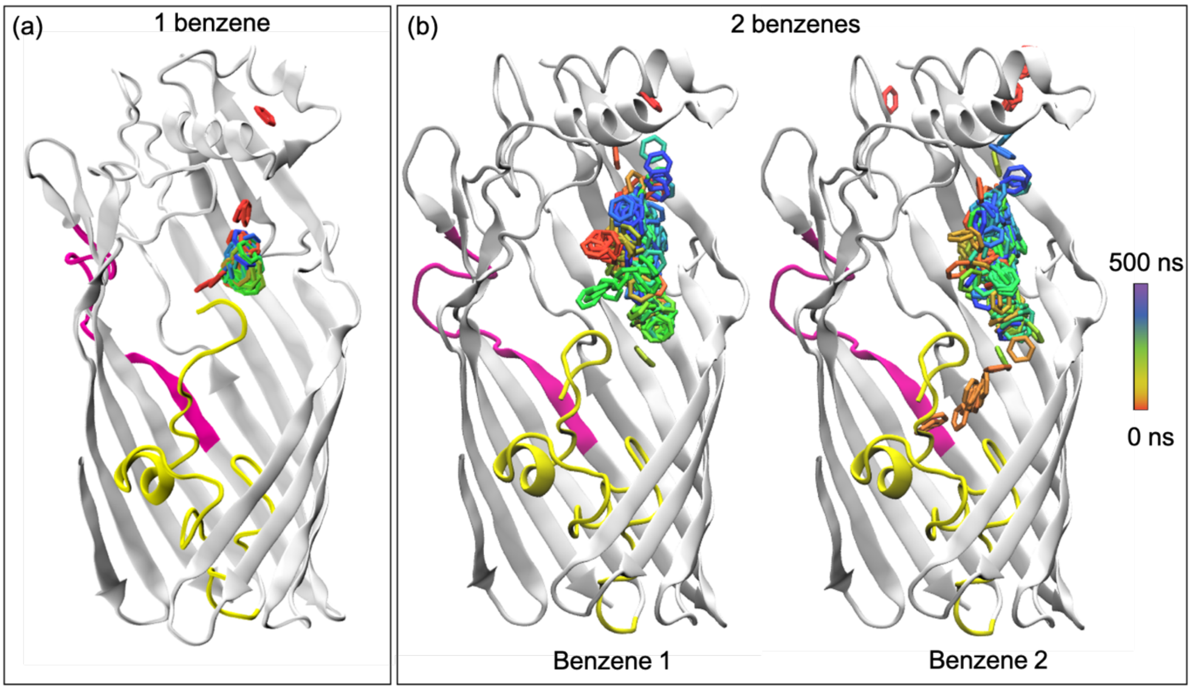

Supplementary Figure 2 Benzene permeation through TodX during two independent equilibrium MD simulations. **a**, Confinement in the P-pocket of the single benzene that entered the protein during this simulation. **b**, Movement of two benzene molecules that entered the protein during this simulation. While the benzene molecules again have an overall preference for the P-pocket, the confinement is not nearly so pronounced as in the simulation shown in (**a**). The N-terminal plug domain of TodX is coloured yellow and the strand S2 is highlighted in pink.

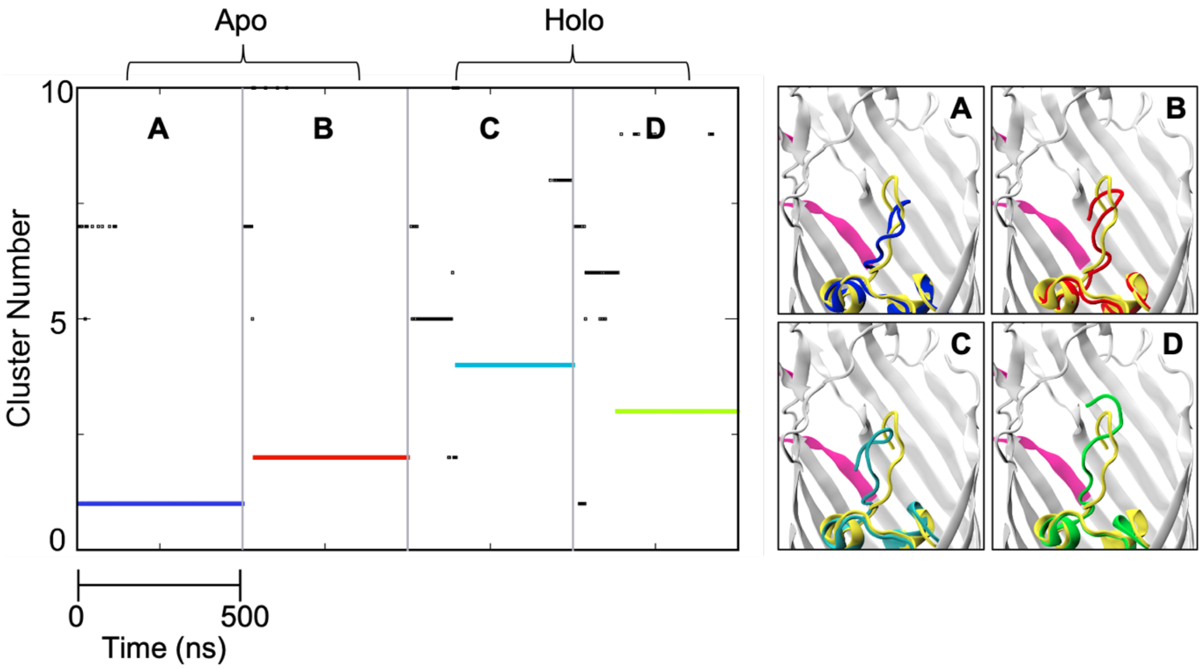

Supplementary Figure 3 Cluster analysis of 4 x 500 ns equilibrium MD simulations (2 apo (A and B) and 2 with benzene (B and C)). In each simulation one cluster dominates, indicated by coloured lines. The conformations of the N-terminus (backbone of residues 1-12) from these dominant clusters are shown in the 4 panels on the right-hand side. The N-terminus in the X-ray crystal structure of TodX is shown in yellow. Panel D corresponds to the conformation (shown in green) that coincides with benzene confinement in the P-pocket. We have termed this ‘state II’.

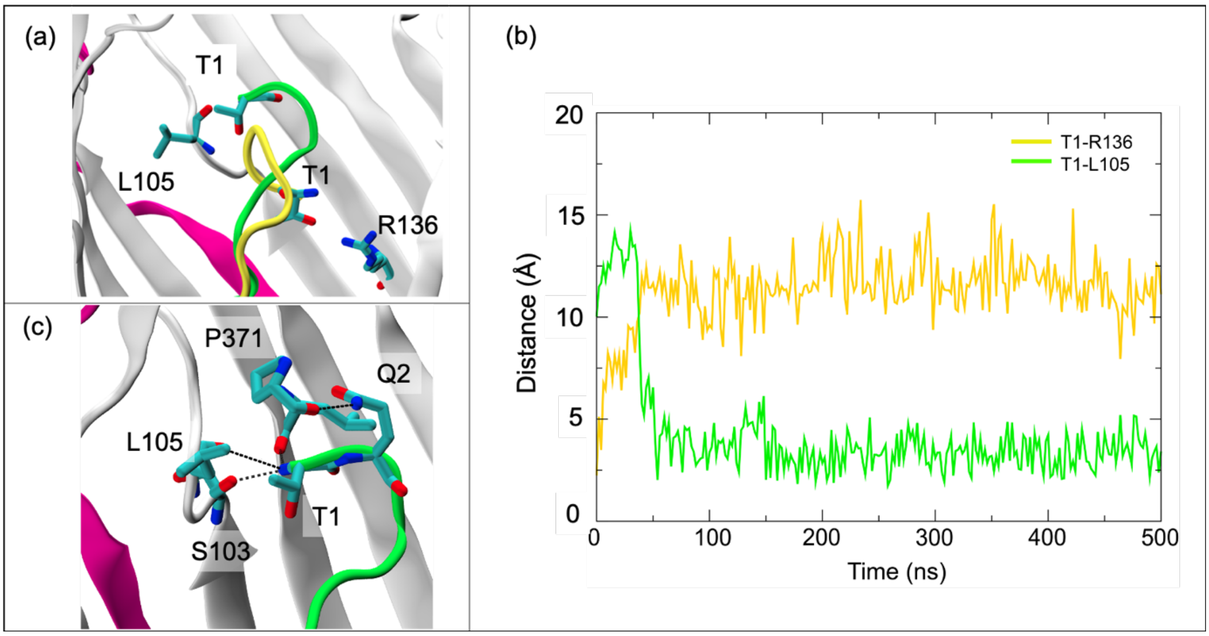

Supplementary Figure 4 N-terminal conformational rearrangement leading to state II. **a**, Conformations of the N terminus in the crystal structure (state I, yellow) and state II (green). Residues Thr1, Leu105 and Arg136 are labelled to highlight the conformational difference between the two states. **b**, Minimum distance between Thr1 and Leu105/Arg136 as a function of simulation time; the conformational change to state II occurs at about 50 ns, leading to a shift in proximity of the N-terminus from Arg136 to Leu105, **c,** interactions that stabilise the N-terminus in state II.

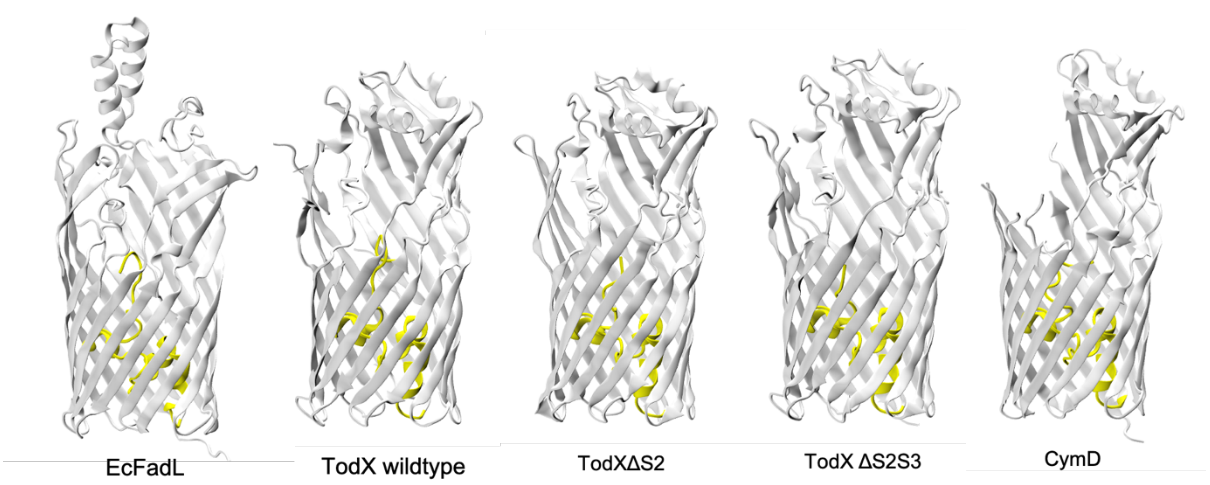

Supplementary Figure 5. X-ray crystal structures of TodX ΔS2, TodX ΔS2S3 and CymD. The structures of EcFadL (PDB ID 1T16) and wild type TodX (PDB ID 3BRZ) are shown for comparison. Missing residues are 1-3 and 401-407 in TodX ΔS2, 1-3 and 400-408 in TodX ΔS2S3, and 1-2, 60-74, 109-126, 366-372, and 405-418 in CymD.

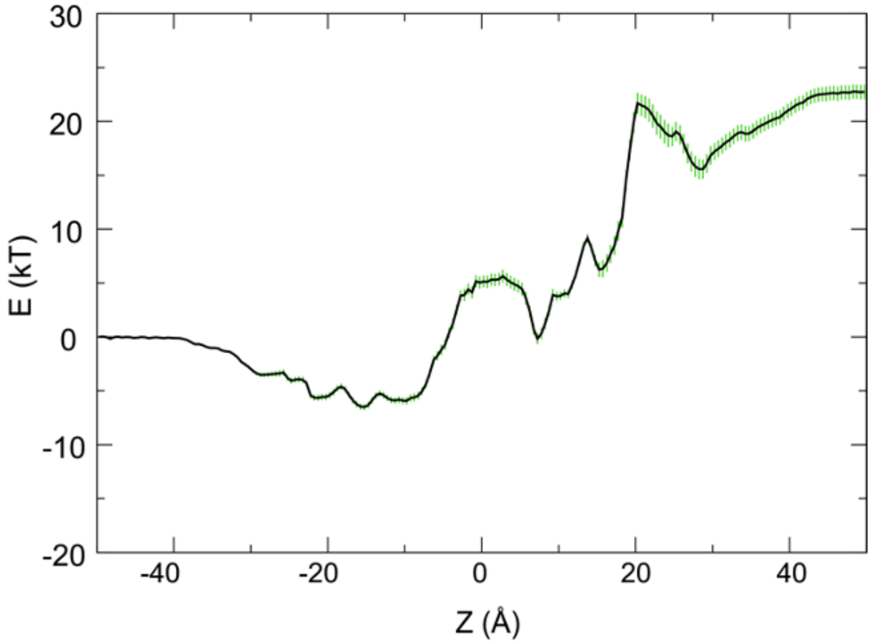

Supplementary Figure 6. The non-cyclised PMF profile of benzene through the classical route of TodX. Standard deviations are shown in green.

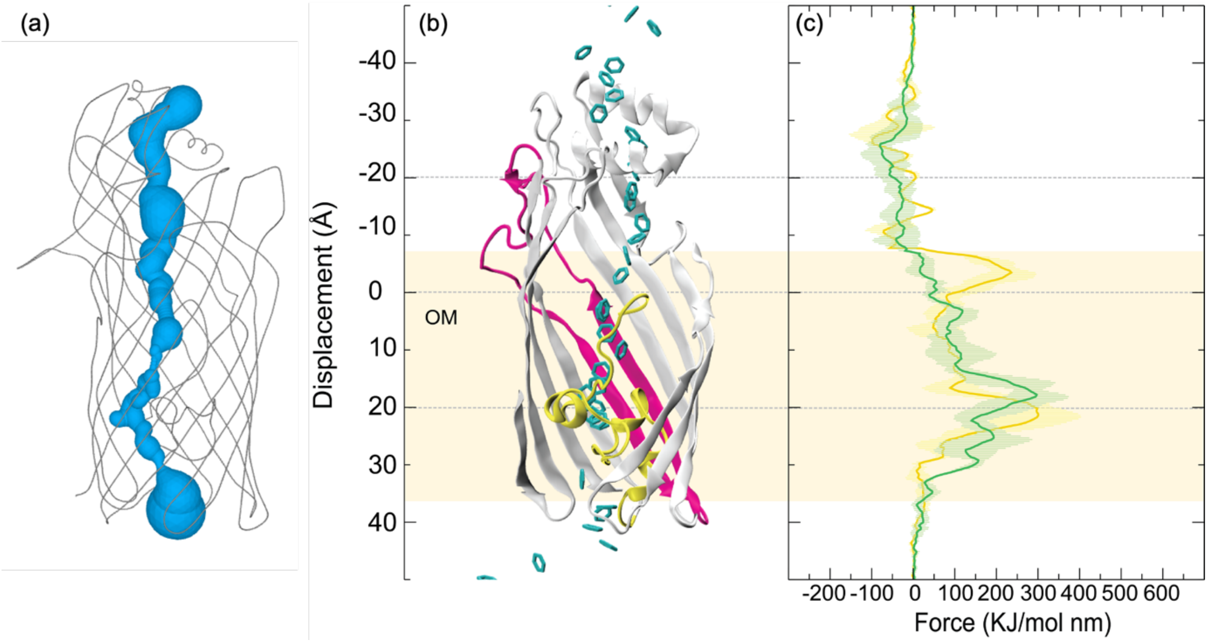

Supplementary Figure 7 Force profiles from steered MD simulations through the classical pathway. **a**, Surface model of the classical channel through TodX based on the crystal structure (3BRZ) after addition of hydrogens. **b**, Snapshots from steered MD simulations of benzene pulled through TodX when the protein is unrestrained in state I. **c**, Force profiles of benzene pulled along the classical route when the protein is unrestrained in state I (yellow), and when the N-terminal plug domain is restrained in state II (green).

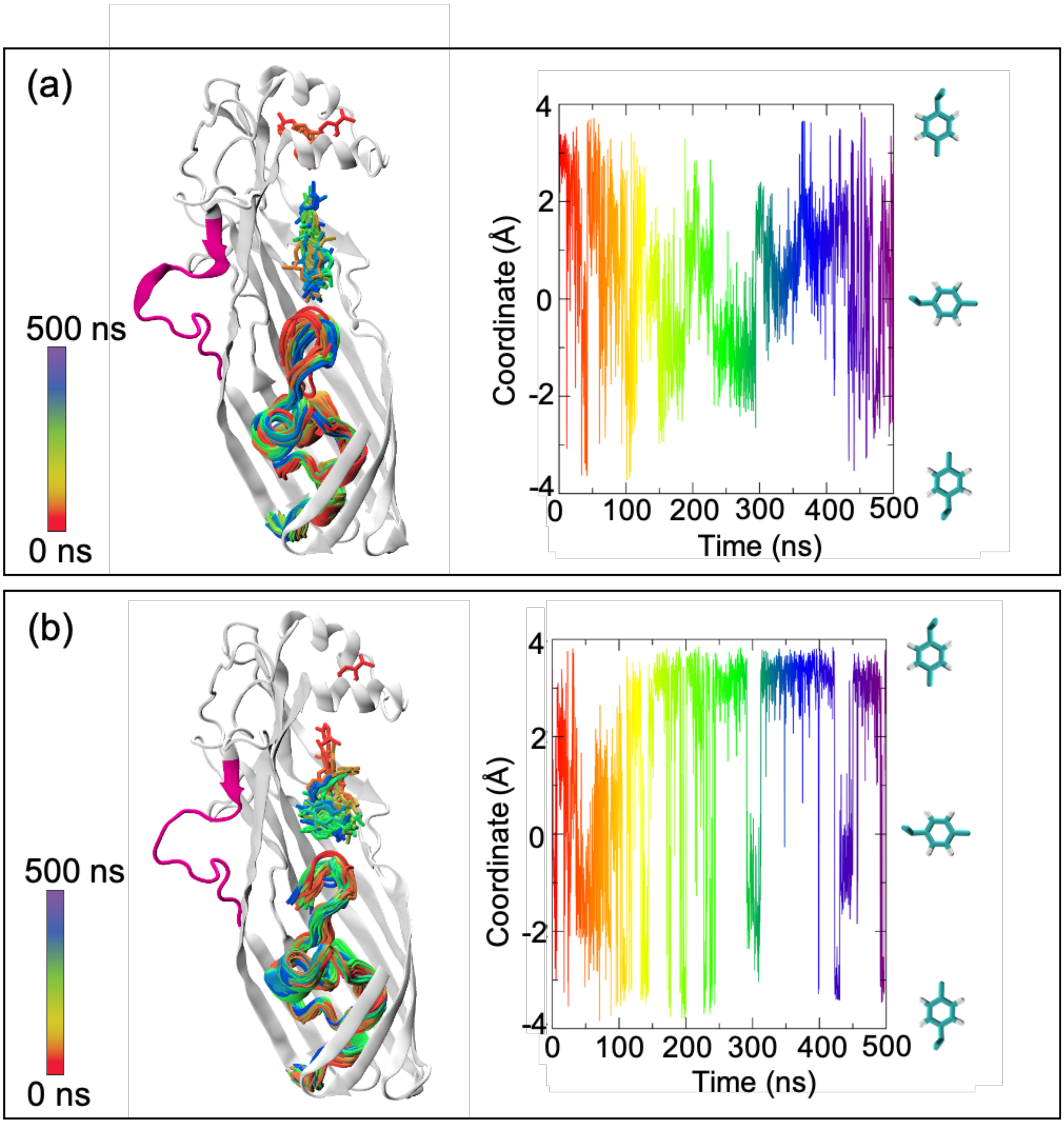

Supplementary Figure 8 Equilibrium MD simulations of p-cymene and CymD. **a, b** Each panel shows one of the independent, 500 ns simulations. In each case, p-cymene remains within the P-pocket. The right-side plots show the orientations adopted by p-cymene throughout the simulations as a function of time. The z-coordinate of the centre of mass of the isopropyl group with respect to the centre of the mass of the 6-membered ring is plotted on the y-axis, with accompanying images of p-cymene to facilitate interpretation. Across the two simulations, there is no single preferred orientation.

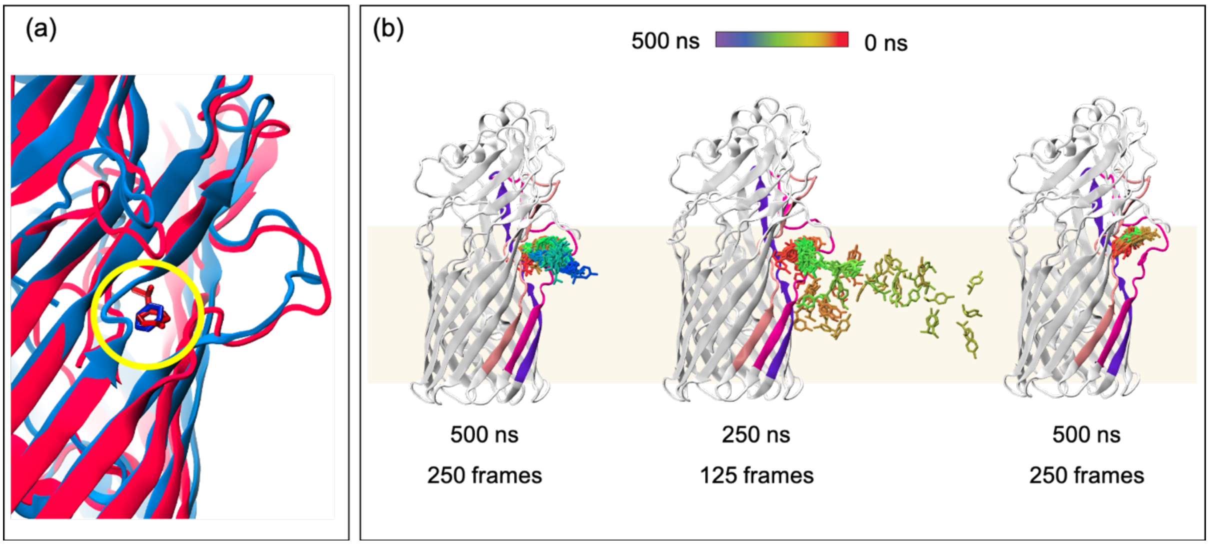

Supplementary Figure 9 Equilibrium MD simulations with p-cymene initially placed at the lateral opening. **a**, Superposition of TodX and benzene (blue) and CymD and p-cymene (red), with the location of the MAH highlighted by the yellow circle. Thus, the equilibrium simulations exploring substrate diffusion into the OM are initiated from equivalent protein-substrate configurations. **b**, Lateral diffusion of p-cymene into the OM within 250 ns in 3 independent simulations.

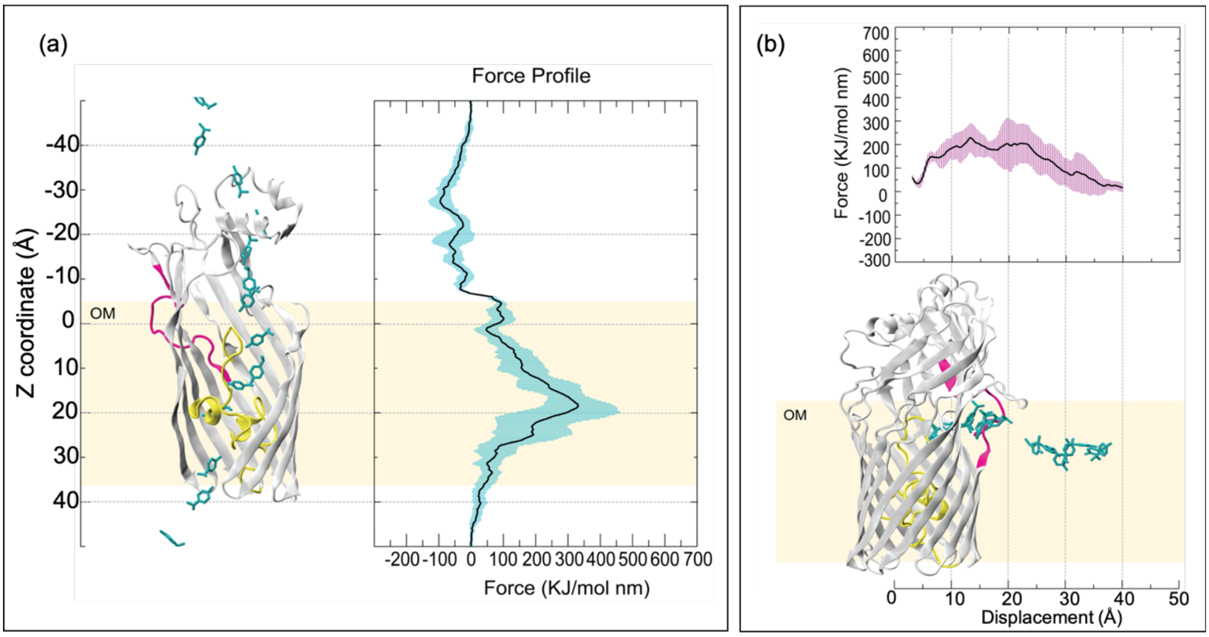

Supplementary Figure 10 Force profiles from steered MD simulations. **a**, Force profile for p-cymene being pulled along the classical route in CymD. There is a large barrier at Z ~20 Å which corresponds to the plug domain. **b**, Force profile for pulling p-cymene through the lateral opening. Only one pathway was identified, for which the profile is similar to that for benzene through TodX via pathway B (Fig. 7). Standard deviations are indicated in blue on panel A and in purple in panel B.

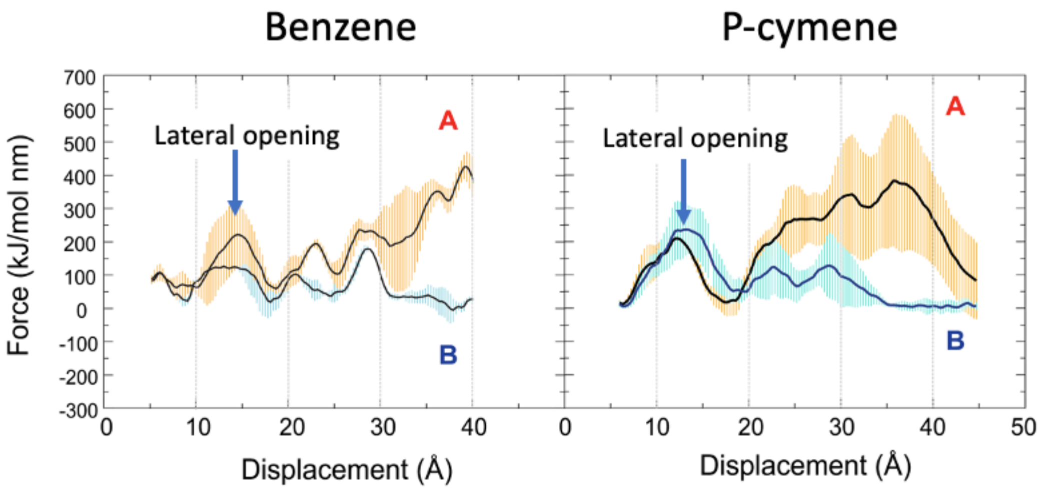

Supplementary Figure 11 Comparison of benzene and p-cymene force profiles when they are pulled out of the lateral opening of TodX. Based on this analysis, MAH exit via pathway B is easiest, with the least force required.

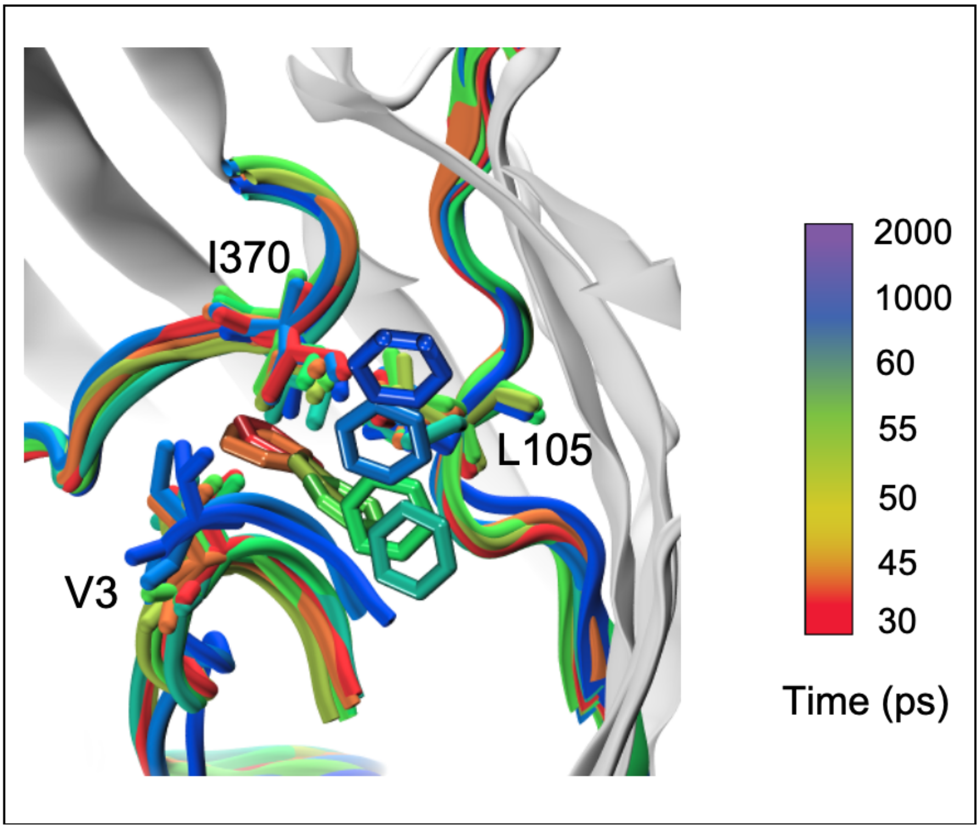

Supplementary Figure 12. Movement of benzene from the lateral opening to the P-pocket and concomitant conformational change during an equilibrium MD simulation initiated from benzene at the lateral opening (red). The timeframe shows that the movement occurs very quickly within a 30 ps timeframe. Thereafter there is less movement as the benzene remains confined to the P-pocket. The side chains of the aliphatic gate residues Val3, Leu105 and Ile370 are shown as stick models.

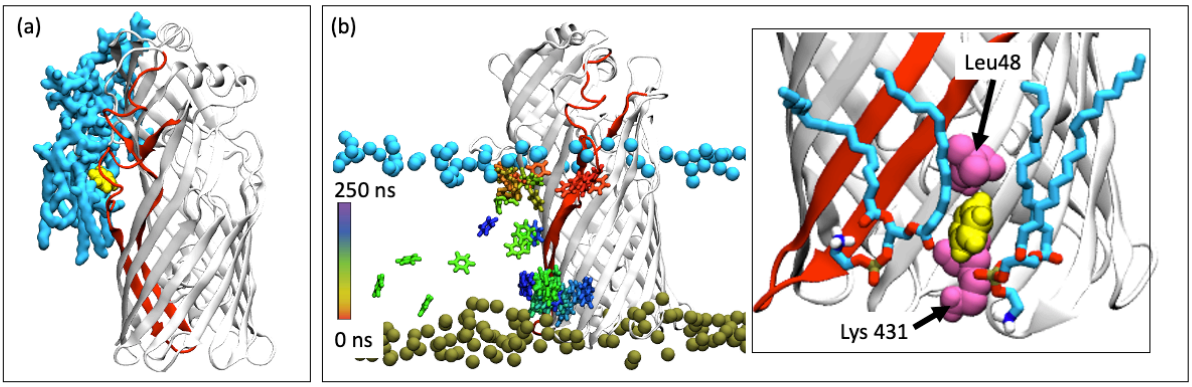

Supplementary Figure 13. The fate of benzene in the OM after emergence from TodX in equilibrium MD simulations. **a**, Cartoon showing the two LPS molecules within 6 Å of benzene, about 20 ns after benzene (yellow) has emerged from the protein. **b**, The benzene location at every 4 ns during a 250 ns simulation is shown. The benzene is very mobile within the OM and even reaches the inner phospholipid leaflet. Benzene becomes confined for around 80 ns at one particular region in one of the simulations, where a pocket is formed by Leu48 and Lys431. This is shown in the inset in panel **b.** Two phospholipids are shown for context regarding location within the OM. The phosphate groups of lipid A (cyan) and phospholipids (olive) are shown as VDW spheres. S2 and S3 are coloured red to show the location of the lateral opening.

Supplementary Table 1 Data collection and refinement statistics (molecular replacement)

	TodX ΔS2<br>PDB 6Z37	TodX ΔS2S3<br>PDB 6Z38	CymD<br>PDB 6Z34
**Data collection**#			
Space group	I222	I222	I222
Cell dimensions			
a, *b*, *c* (Å)	80, 116, 170	80, 116, 173	78, 88,133
α, β, γ (°)	90, 90, 90	90, 90, 90	90, 90, 90
Resolution (Å)	48.1-3.05<br>(3.26-3.05)*	48.1-2.90<br>(3.08-2.90)	43.8-2.27<br>(2.31-2.27)
R_{merge}	8.2 (48.9)	7.9 (138)	7.6 (125)
$I / \sigma I$	11.5 (2.0)	13.2 (1.4)	15.0 (1.1)
Completeness (%)	100 (100)	100 (100)	98.6 (84.9)
Redundancy	7.3 (7.6)	7.4 (7.7)	6.8 (4.7)
**Refinement**			
Resolution (Å)	48.1-3.05	48.1-2.90	43.8-2.27
No. reflections	15,545	18,252	21,316
R_{work} / R_{free} (%)	23.1/29.8	25.3/31.8	21.2/27.1
No. atoms			
Protein	3146	3115	2886
Water	-	-	13
B-factors			
Protein	128	128	76
Water	-	-	54
R.m.s. deviations			
Bond lengths (Å)	0.010	0.010	0.008
Bond angles (°)	1.34	1.33	1.07

# One crystal was used for each data collection.

* Values in parentheses are for highest-resolution shell.

Supplementary Table 2 Summary of the equilibrium MD simulation systems

System	Notes	Substrate	Simulation length (ns)
Apo TodX		-	500 ($\times$ 2)
Holo TodX	Benzenes initially placed near extracellular mouth of TodX	3 Benzene	500 ($\times$ 2)
TodX Lateral	Benzene initially placed at lateral opening	1 Benzene	250 ($\times$ 6)
Apo CymD		-	500 ($\times$ 2)
Holo CymD	p-cymenes initially placed near extracellular mouth of CymD	3 P-cymene	500 ($\times$ 2)
CymD Lateral	p-cymene initially placed at lateral opening	1 P-cymene	250 ($\times$ 6)

Supplementary References

1. Keane, A., Lau, P.C. & Ghoshal, S. Use of a whole-cell biosensor to assess the bioavailability enhancement of aromatic hydrocarbon compounds by nonionic surfactants. *Biotechnol Bioeng*. **99**, 86-98 (2008).
2. Wang, Y., Rawlings, M., Gibson, D.T., Labbé, D., Bergeron, H., Brousseau, R. & Lau, P.C. Identification of a membrane protein and a truncated LysR-type regulator associated with the toluene degradation pathway in Pseudomonas putida F1. *Mol Gen Genet* **246**, 570-579 (1995).
