# Supplementary material for: Uptake of monoaromatic hydrocarbons during biodegradation by FadL channel-mediated lateral diffusion
Source: Nat Commun. 2020 Dec 10;11:6331. doi: 10.1038/s41467-020-20126-y (PMC7728783; doi:10.1038/s41467-020-20126-y)
Supplement: Supplementary file 3 — Supplementary Movie Description [file 41467_2020_20126_MOESM3_ESM.pdf]

Description of Additional Supplementary Information:

Title: Supplementary Movie

Description: Diffusion pathway for benzene through TodX. To generate the movie, equilibrium MD simulations were combined with steered MD simulations.
