# Supplementary material for: Uptake of monoaromatic hydrocarbons during biodegradation by FadL channel-mediated lateral diffusion
Source: Nat Commun. 2020 Dec 10;11:6331. doi: 10.1038/s41467-020-20126-y (PMC7728783; doi:10.1038/s41467-020-20126-y)
Supplement: Supplementary file 5 — Reporting Summary [file 41467_2020_20126_MOESM5_ESM.pdf]

Corresponding author(s): Syma Khalid, Bert van den Berg

Last updated by author(s): 28 October 2020

Reporting Summary

Nature Research wishes to improve the reproducibility of the work that we publish. This form provides structure for consistency and transparency in reporting. For further information on Nature Research policies, see our Editorial Policies and the Editorial Policy Checklist.

Statistics

For all statistical analyses, confirm that the following items are present in the figure legend, table legend, main text, or Methods section.

n/a	Confirmed	
☐	☒	The exact sample size (*n*) for each experimental group/condition, given as a discrete number and unit of measurement
☐	☒	A statement on whether measurements were taken from distinct samples or whether the same sample was measured repeatedly
☐	☒	The statistical test(s) used AND whether they are one- or two-sided<br>*Only common tests should be described solely by name; describe more complex techniques in the Methods section.*
☒	☐	A description of all covariates tested
☒	☐	A description of any assumptions or corrections, such as tests of normality and adjustment for multiple comparisons
☐	☒	A full description of the statistical parameters including central tendency (e.g. means) or other basic estimates (e.g. regression coefficient) AND variation (e.g. standard deviation) or associated estimates of uncertainty (e.g. confidence intervals)
☐	☒	For null hypothesis testing, the test statistic (e.g. F, t, r) with confidence intervals, effect sizes, degrees of freedom and P value noted<br>*Give P values as exact values whenever suitable.*
☒	☐	For Bayesian analysis, information on the choice of priors and Markov chain Monte Carlo settings
☒	☐	For hierarchical and complex designs, identification of the appropriate level for tests and full reporting of outcomes
☒	☐	Estimates of effect sizes (e.g. Cohen's d, Pearson's r), indicating how they were calculated

Our web collection on statistics for biologists contains articles on many of the points above.

Software and code

Policy information about availability of computer code

Data collection	Addition of missing residues to X-ray structures: Modeller 9.02<br>Molecular Dynamics simulations and analyses: GROMACS 5.1.4<br>Visualisation and analyses: VMD version 1.9.4 & PYMOL 2.4.0a0.
Data analysis	X-ray data processing and refinement:<br>Xia2 0.6.467<br>DIALS 3.1<br>Pointless 1.12.2<br>Aimless 0.7.4<br>Phenix version 1.18-3855 (includes Phaser)<br>Coot 0.9<br><br>Making figures: GraphPad Prism version 7.

For manuscripts utilizing custom algorithms or software that are central to the research but not yet described in published literature, software must be made available to editors and reviewers. We strongly encourage code deposition in a community repository (e.g. GitHub). See the Nature Research guidelines for submitting code & software for further information.

Data

Policy information about availability of data

All manuscripts must include a data availability statement. This statement should provide the following information, where applicable:

- Accession codes, unique identifiers, or web links for publicly available datasets
- A list of figures that have associated raw data
- A description of any restrictions on data availability

The data supporting the findings of this study, including simulation snapshots and parameters, are available from the corresponding authors upon reasonable request. Coordinates and structure factors that support the findings of this study have been deposited in the Protein Data Bank with accession codes 6Z34 (CymD), 6Z37 (TodX deltaS2) and 6Z38 (TodX deltaS2S3). The coordinates of TodX and CymD with the missing parts modelled have been deposited in Zenodo. Source data are provided with this paper.

Field-specific reporting

Please select the one below that is the best fit for your research. If you are not sure, read the appropriate sections before making your selection.

☒ Life sciences ☐ Behavioural & social sciences ☐ Ecological, evolutionary & environmental sciences

For a reference copy of the document with all sections, see nature.com/documents/nr-reporting-summary-flat.pdf

Life sciences study design

All studies must disclose on these points even when the disclosure is negative.

Sample size	No statistical methods were used to predetermine sample size. In all cases, a total of 5-10 of the isolated, largest colonies at the front of the streak from 2 different plates were used for colony size determination. In all cases this resulted in standard deviations of ~10% or smaller.
Data exclusions	No data exclusion was needed.
Replication	For plate visualisation, the replicas of 2 or 3 independent growth experiments gave similar results. Colony sizes were reported from 2 independent growth experiments.
Randomization	This is not relevant to our study, because no grouping was needed.
Blinding	Investigators were not blinded to group allocation, as no grouping was needed for this study.

Reporting for specific materials, systems and methods

We require information from authors about some types of materials, experimental systems and methods used in many studies. Here, indicate whether each material, system or method listed is relevant to your study. If you are not sure if a list item applies to your research, read the appropriate section before selecting a response.

Materials & experimental systems

n/a	Involved in the study	
☒	☐	Antibodies
☒	☐	Eukaryotic cell lines
☒	☐	Palaeontology and archaeology
☒	☐	Animals and other organisms
☒	☐	Human research participants
☒	☐	Clinical data
☒	☐	Dual use research of concern

Methods

n/a	Involved in the study	
☒	☐	ChIP-seq
☒	☐	Flow cytometry
☒	☐	MRI-based neuroimaging
